# Cancer‐directed surgery in patients with metastatic cancer: A systematic review and meta‐analysis of randomized evidence

**DOI:** 10.1002/cam4.6061

**Published:** 2023-06-13

**Authors:** Mary Kathryn Abel, Ellen L. Myers, Ellen Minkin, Peggy Tahir, Alex B. Haynes, Jason D. Wright, J. Alejandro Rauh‐Hain, Alexander Melamed

**Affiliations:** ^1^ Division of Gynecologic Surgery, Department of Obstetrics and Gynecology Massachusetts General Hospital Boston Massachusetts USA; ^2^ Department of Obstetrics and Gynecology, Christiana Hospital Newark Delaware USA; ^3^ University of Southern California Los Angeles California USA; ^4^ Library, University of California, San Francisco San Francisco California USA; ^5^ Department of Surgery and Perioperative Care, Dell Medical School The University of Texas at Austin Austin Texas USA; ^6^ Division of Gynecologic Oncology, Department of Obstetrics and Gynecology Columbia University Vagelos College of Physicians and Surgeons, New York‐Presbyterian Hospital New York New York USA; ^7^ Department of Gynecologic Oncology and Reproductive Medicine The University of Texas MD Anderson Cancer Center Houston Texas USA

**Keywords:** cancer, chemotherapy, metastatic, stage IV, surgery

## Abstract

**Purpose:**

To assess the impact of primary‐site surgery plus systemic therapy compared to systemic therapy alone on overall survival in common metastatic cancer types.

**Methods:**

Data sources included Embase, PubMed, and Web of Science (January 1, 1995–March 22, 2023). Randomized controlled trials were included that enrolled patients diagnosed with the 10 most common de novo metastatic cancer types in the Surveillance, Epidemiology, and End Results database and randomized patients to resection of the primary site and systemic therapy versus systemic treatment alone. Random‐effects models were used to pool associations by cancer type.

**Results:**

Eight studies with 1774 patients evaluating the efficacy of surgery in breast, renal, stomach, and colorectal cancer were included. There was no statistically significant reduction in risk of all‐cause mortality associated with surgical intervention for metastatic breast (HR = 0.94, 95% CI 0.63–1.40) or renal cancer (HR = 0.79, 95% CI 0.53–1.20), although results were heterogeneous (*I* ^2^ = 73.7% and 80.6%, respectively). One study evaluating gastrectomy in metastatic stomach cancer found no benefit (HR = 1.09, 95% CI 0.78–1.52), while a small trial suggested that surgery and hyperthermic intraperitoneal chemotherapy might be beneficial for colorectal cancer with peritoneal metastasis (HR = 0.55, 95% CI 0.32–0.95).

**Conclusions:**

Few randomized trials have evaluated cancer‐directed surgery among patients with metastatic solid malignancies.

## 1 INTRODUCTION

Surgery was the first effective treatment for malignant neoplasms,^1^ and it continues to be an important treatment modality for most solid malignancies. Although surgery is used nearly universally in the treatment of localized tumors, its role is less clear in the metastatic setting when cure is unlikely. While surgery may play an important therapeutic role for some patients with metastatic colorectal,^2^, ^3^ renal,^4^ and ovarian cancer,^5^ it is almost always avoided in metastatic lung,^6^ pancreatic,^7^ and prostate cancer.^8^ Furthermore, there appear to be discrepancies between evidence and real‐world practice. For example, even after the publication of two randomized trials that demonstrated an overall survival advantage for cytoreductive nephrectomy and interferon therapy compared to interferon therapy alone in patients with metastatic renal cell carcinoma,^9^, ^10^ there was a paradoxical decrease in the utilization of the procedure in the United States.^8^

Metastatic cancer is responsible for a large global burden of morbidity and mortality, contributing to 66.7%–90% of all cancer deaths.^8^, ^11^, ^12^ For most cancers, the presence of metastatic disease at the time of diagnosis is associated with a dire prognosis, and effective treatments are urgently needed. Resection of the primary tumor may benefit patients with metastatic disease by improving host anticancer immune response,^13^ removing cells that facilitate further invasion and metastasis,^14^ and enhancing the effectiveness of systemic therapy.^15^ While studies have synthesized clinical evidence on the efficacy of primary site surgery for metastatic breast,^16^ renal,^17^ and colon cancer,^18^ many have included observational studies which are likely to be biased. Furthermore, these studies have not examined the evidence supporting primary site surgery for other commonly encountered metastatic cancers.

In this study, we seek to provide a broad understanding of available experimental evidence evaluating the effectiveness of cancer‐directed surgery among patients with metastatic cancer and to characterize the utilization of surgery across cancer types. To this end, we conducted a systematic review and meta‐analysis of randomized trials evaluating the efficacy of primary site surgery on overall survival among the 10 most common metastatic cancer types diagnosed in the United States. Furthermore, we describe how often such operations are undertaken in each of these cancer types using data from a population‐based cancer registry. Finally, we evaluate whether the frequency of primary‐site surgery in the metastatic setting is associated with the availability of randomized evidence to support its use.

## 2 METHODS

### 2.1 Literature search

In this systematic review and meta‐analysis, we conducted a comprehensive database search regarding surgery for metastatic cancer in Embase (Elsevier), PubMed (National Library of Medicine), and Web of Science (Clarivate Analytics). A medical librarian (P.T.) who specializes in systematic reviews developed the search, which was broken down into five main concepts that were subsequently combined: cancer surgeries, specific surgical procedures, metastatic concepts, survival analysis and mortality, and randomized controlled trials. Both index terms (Mesh or Emtree) were used in addition to keywords when constructing the searches, as appropriate to the specific database. The search was restricted to studies published between January 1, 1995, and March 22, 2023, that were written in English and included patients who were diagnosed with one of the 10 most common metastatic cancers in the Surveillance, Epidemiology, and End Results Program (SEER) database (including lung, pancreas, colon, breast, prostate, ovary, stomach, kidney, rectum, and esophagus). The complete searches for each database are listed in Table S1.

Our findings are reported in accordance with the Preferred Reporting Items for Systematic Review and Meta‐Analyses (PRISMA) guidelines for systematic reviews of clinical trials.^19^ The study protocol is registered in PROSPERO (CRD42020194425).

### 2.2 Study selection

Title and abstracts of the articles were reviewed independently by two authors (M.K.A. and E.M.) to identify potentially relevant clinical trials. A third author (A.M.) reviewed the included title and abstracts to resolve any disagreements and remove any articles that were included erroneously. Studies that were included after title and abstract review were then retrieved for full text review by the same two authors (M.K.A. and E.M.) and reviewed with inclusion and exclusion criteria. Again, any disagreements were resolved by a third author (A.M.). Additionally, references of included articles and systematic reviews pertinent to the research question were evaluated to find other potential articles.

### 2.3 Eligibility criteria and data extraction

Randomized controlled trials were included in the systematic review and meta‐analysis if they met the following criteria: (1) enrolled patients with metastatic cancers at the time of diagnosis (i.e., spread to distant lymph nodes and/or organs), including solid tumors of the lung, pancreas, ovary, breast, prostate, stomach, kidney, colon, esophagus, and rectum, (2) randomized patients to cancer resection with systemic treatment compared with systemic treatment alone, (3) compared overall survival between the study arms, and (4) reported a median follow‐up time of at least 6 months.

Non‐English studies and studies other than randomized controlled trials reported in a peer‐reviewed journal (such as observational studies, conference abstracts, systematic review or meta‐analyses, or randomized controlled trial protocols without data) were excluded. Randomized clinical trials that did not report overall survival outcomes, included only patients with locally advanced cancer, and/or randomized patients to surgery that did not include resection of the primary tumor were also excluded. Finally, when multiple reports from the same study were identified, we included the most up‐to‐date report that included all required data by our data abstraction instrument.

A standardized data abstraction instrument was used to extract data from included studies. Studies were categorized by location of primary tumor. Extracted variables included study characteristics (dates of enrollment, inclusion and exclusion criteria, treatment arms, primary and secondary endpoints), mortality outcomes (number of deaths and median overall survival by study arm, hazard ratios [HR] and 95% confidence intervals [CI] for all‐cause mortality), and median follow‐up time. All hazard ratios were recorded with systemic therapy only as the referent group. When no hazard ratio was reported, we estimated the hazard ratio based on the number of deaths and the p‐value from the log‐rank using the method described by Tierney et al.^20^ Two authors (M.K.A. and E.M.) independently performed the data extraction, and data were validated by a third author (A.M.).

### 2.4 Study quality assessment

The quality of each randomized controlled trial included in our systematic review and meta‐analysis was assessed using the Revised Cochrane Risk‐of‐Bias Tool for Randomized Trials (RoB2).^21^ This tool assesses bias using questions divided into the following five domains: (1) risk of bias arising from the randomization process, (2) risk of bias due to deviations from the intended interventions (effect of assignment to intervention), (3) risk of bias due to missing outcome data, (4) risk of bias in measurement of the outcome, and (5) risk of bias in selection of the reported result. The RoB2 algorithms were used to determine whether studies were at low, some, or high risk for bias in each domain with respect to the outcome of overall survival. E.M. independently scored each study, and two authors (M.K.A. and A.M.) evaluated the scoring post‐hoc. Using scores from each domain, overall risk of bias was then categorized as low (low risk of bias for all domains), some (some risk in at least one domain but not high risk in any domain), or high (high risk in at least one domain or some concerns for multiple domains in a way that substantially lowers confidence in the result).

### 2.5 Utilization of primary site surgery in metastatic cancer in the United States

Using data from the SEER's 18 registry research data (November 2018 submission), we identified all patients diagnosed with a solid organ malignancy between 2012 and 2016 who had metastatic disease (defined as AJCC 7th edition M stage of 1, 1A, 1B, 1C, 1D, or 1 not otherwise specified). The period between 2012 and 2016 represented the most recent 5 years for which SEER data were available at the time of the analysis. We then ranked disease sites by the number of patients diagnosed with metastatic cancer during the study period and restricted our analysis to the 10 most common disease sites to account for the greatest share of cases of metastatic cancer. For each site, we defined primary site surgery as an excisional procedure of the primary site (Table S2) and calculated the proportion of patients who underwent such a procedure.

### 2.6 Statistical analysis

When more than one randomized trial was identified for a cancer type, we estimated a pooled all‐cause mortality hazard ratio using a random‐effects model. We selected a random‐effects model to allow for difference in study populations and periods. Heterogeneity was assessed using the *I* ^2^ statistic, with 0%–40%, 30%–60%, 50%–90%, and 75%–90% *I* ^2^ scores representing possibly unimportant, moderate, substantial, and considerable heterogeneity, respectively.^22^ We estimated median survival for each cancer type from SEER data using the Kaplan–Meier method. Hypothesis tests were 2‐sided with a significance threshold of 0.05. Statistical analyses were performed in Stata Version 16.0 (StataCorp LLC).

## 3 RESULTS

### 3.1 Study selection and quality of studies

**FIGURE 1:**
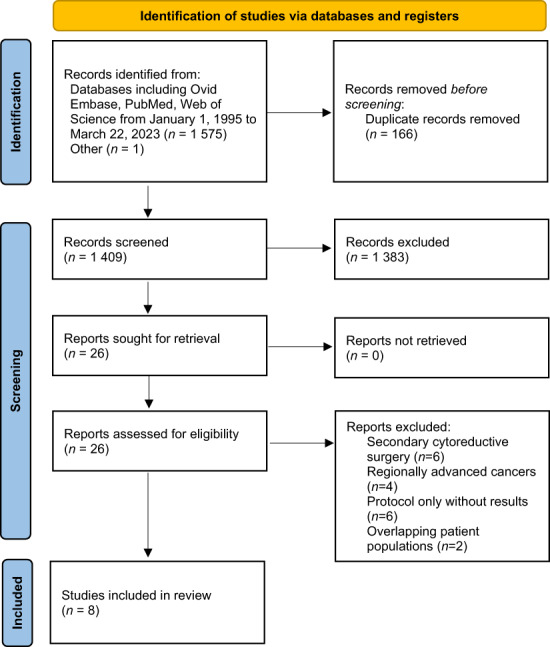
Preferred Reporting Items for Systematic Reviews and Meta‐Analyses (PRISMA) flow diagram.^21^

The initial search of the databases identified a total of 1576 citations, with 1409 unique reports included after duplicate manuscripts and non‐English language studies were removed. Of those screened, a total of 26 reports were evaluated for inclusion and exclusion criteria. Six reports that described studies of secondary cytoreductive surgery in ovarian cancer treatment were removed.^23^, ^24^, ^25^, ^26^, ^27^, ^28^ Four reports were excluded because they included patients with regionally advanced cancers but non‐metastatic cancers.^29^, ^30^, ^31^, ^32^ Six publications reported study protocols only.^33^, ^34^, ^35^, ^36^, ^37^, ^38^ Finally, two studies included overlapping populations, and the follow‐up studies were excluded because they omitted several variables of interest that were available in the primary papers.^39^, ^40^, ^41^, ^42^ Figure 1 shows the PRISMA flowchart for the entire study selection process at each stage.

Data quality and risk of bias were assessed in the eight remaining articles using RoB2 (Table S3). Of the studies that were included in our analysis, seven had low overall risk of bias. One study was at some overall risk of bias stemming from either deviation from the intended intervention or selection of reported results.^44^ Because no studies were deemed to be high overall risk for bias, all studies remained in the analysis.

### 3.2 Included studies

We identified eight randomized clinical trials that met criteria for inclusion in the systematic review and meta‐analysis.^9^, ^10^, ^39^, ^42^, ^43^, ^44^, ^45^, ^46^ Table 1 tabulates study arms, number of patients and deaths, median follow‐up time, and survival data. Three trials evaluated the effect of breast surgery on survival among patients with metastatic breast cancer.^42^, ^43^, ^44^ Another three trials, compared cytoreductive nephrectomy and systemic therapy versus systemic therapy alone in patients with renal cell carcinoma.^9^, ^10^, ^45^ An additional study assigned patients with metastatic gastric cancer to gastrectomy (with or without resection of metastatic lesions) followed by chemotherapy or chemotherapy only.^46^ Finally, one study evaluated the effect of extensive cytoreductive surgery and intraoperative hyperthermic intraperitoneal chemotherapy (HIPEC) followed by intravenous chemotherapy compared to intravenous chemotherapy only in patients with metastatic colon or rectal cancer.^39^

**TABLE 1:** High‐quality studies included in systematic review.

First author, year	Cancer site	Intervention	Total number of patients	Number of patients per arm	Number of deaths per arm	Hazard ratio (95% CI)^a^	Median follow‐up (months)	Median overall survival per arm (months)
Badwe 2015	Breast	Surgery arm: Surgical therapy with or without radiotherapy following systemic therapy No surgery arm: Systemic therapy alone	350	Surgery arm: 173 No surgery arm: 177	Surgery arm: 118 No surgery arm: 117	1.04 (0.81–1.34)	23	Surgery arm: 19.2 No surgery arm: 20.5
Fitzal 2019	Breast	Surgery arm: Surgical therapy (lumpectomy or mastectomy) with axillary staging followed by systemic therapy No surgery arm: Systemic therapy alone	90	Surgery arm: 45 No surgery arm: 45	Surgery arm: NR No surgery arm: NR	1.45 (0.75–2.78)	37.5	Surgery arm: 34.6 No surgery arm: 54.8
Soran 2018	Breast	Surgery arm: Initial locoregional therapy (mastectomy or breast conservation surgery with level I–II axillary clearance; + radiotherapy for patients undergoing breast conservation surgery) followed by systemic therapy No surgery arm: Systemic therapy only	278	Surgery arm: 140 No surgery arm: 138	Surgery arm: 76 No surgery arm: 101	0.66 (0.49–0.88)	Surgery arm: 41 No surgery arm: 37	Surgery arm: 46 No surgery arm: 37
Flanigan 2001	Kidney	Surgery arm: Radical nephrectomy followed by interferon alfa‐2b No surgery arm: Interferon alone	241	Surgery arm: 120 No surgery arm: 121	Surgery arm: 106 No surgery arm: 115	0.71 (0.55–0.93)	12.3	Surgery arm: 11.1 No surgery arm: 8.1
Mickisch 2001	Kidney	Surgery arm: Nephrectomy followed by interferon‐alpha‐based immunotherapy No surgery arm: interferon‐alpha‐based immunotherapy	85	Surgery arm: 42 No surgery arm: 43	Surgery arm: 25 No surgery arm: 30	0.54 (0.31–0.94)	NR	Surgery arm: 17 No surgery arm: 7
Méjean 2018	Kidney	Surgery arm: Cytoreductive nephrectomy followed by sunitinib No surgery arm: Sunitinib alone	450	Surgery arm: 226 No surgery arm: 224	Surgery arm: 165 No surgery arm: 161	1.12 (0.91–1.41)	50.9	Surgery arm: 13.9 No surgery arm: 18.4
Fujitani 2016	Stomach	Surgery arm: Gastrectomy with or without resection of metastatic lesions and chemotherapy No surgery arm: Chemotherapy	175	Surgery arm: 89 No surgery arm: 86	Surgery arm: 73 No surgery arm: 71	1.09 (0.78–1.52)	14.5	Surgery arm: 14.3 No surgery arm: 16.6
Verwaal 2003	Colon	Surgery arm: Aggressive cytoreductive surgery followed by HIPEC, then systemic chemotherapy No surgery arm: Systemic chemotherapy with or without palliative surgery	105	Surgery arm: 54 No surgery arm: 51	Surgery arm: 24 No surgery arm: 31	0.55 (0.32–0.95)	21.6	Surgery arm: 22.4 No surgery arm: 12.6

^a^ Hazard ratio <1 favors surgical treatment.

Abbreviations: CI, confidence interval; NR, not reported.

A total of 1774 patients were enrolled in these trials, including 889 (50.1%) individuals who underwent a cancer‐directed surgical procedure for metastatic cancer and 885 (49.9%) individuals who had systemic therapy alone. Of the 889 individuals who received surgery, 587 died during the study period, while 626 of the 885 individuals who did not receive surgery died, although one study did not report number of deaths by intervention.^43^ The median follow‐up time in months ranged from 12.3 to 50.9 months. Median overall survival time in those who underwent a surgical procedure ranged from 11.1 to 46.0 months, while the median overall survival time in those who did not receive surgery ranged from 7.0 to 54.8 months.

### 3.3 Breast cancer clinical trials

All three breast cancer trials included patients with de novo metastatic breast cancer, although none required biopsy.^42^, ^43^, ^44^ In all of the studies, local treatment of metastatic sites was allowed but not recommended by protocol. Each study compared locoregional treatment of the breast (lumpectomy or mastectomy) and axillary lymph nodes followed by systemic chemotherapy, anti‐HER2 therapy, and/or anti‐hormone therapy with or without radiation based on local standards. The primary endpoint in all studies was overall survival, and study populations ranged from 90 to 350 patients for a total of 718 patients (Table 1). In the largest trial reported by Badwe et al. in 2015,^44^ all patients underwent either pre‐randomization chemotherapy (cyclophosphamide/epirubicin or doxorubicin/fluorouracil, anthracycline plus taxane, paclitaxel plus carboplatin, or HER2‐targeted treatment) or endocrine therapy (aromatase inhibitor or tamoxifen). Patients with operable primary tumors were randomized to surgery with mastectomy or lumpectomy with or without radiation therapy or no surgery or radiotherapy, while those with inoperable breast masses were treated with neoadjuvant chemotherapy and randomized if a complete or partial response was observed. No neoadjuvant therapy was performed in the other trials.^42^, ^43^ In Badwe et al., radiation was administered frequently to patient receiving mastectomy and breast conserving therapy resulting in a locoregional radiotherapy rate of 80%.^44^ By contrast, breast irradiation was completed in only 20% of patients assigned to locoregional treatment in the study reported by Fitzal et al.^43^ The rate of locoregional radiotherapy was not reported by Soran et al.^42^ The distribution of metastatic disease sites also varied among these studies; the proportion of patients with bone‐only metastases was 29%, 38%, and 45% in the trial reported by Badwe et al.,^44^ Fitzal et al.,^43^ and Soran et al.,^42^ respectively.

**FIGURE 2:**
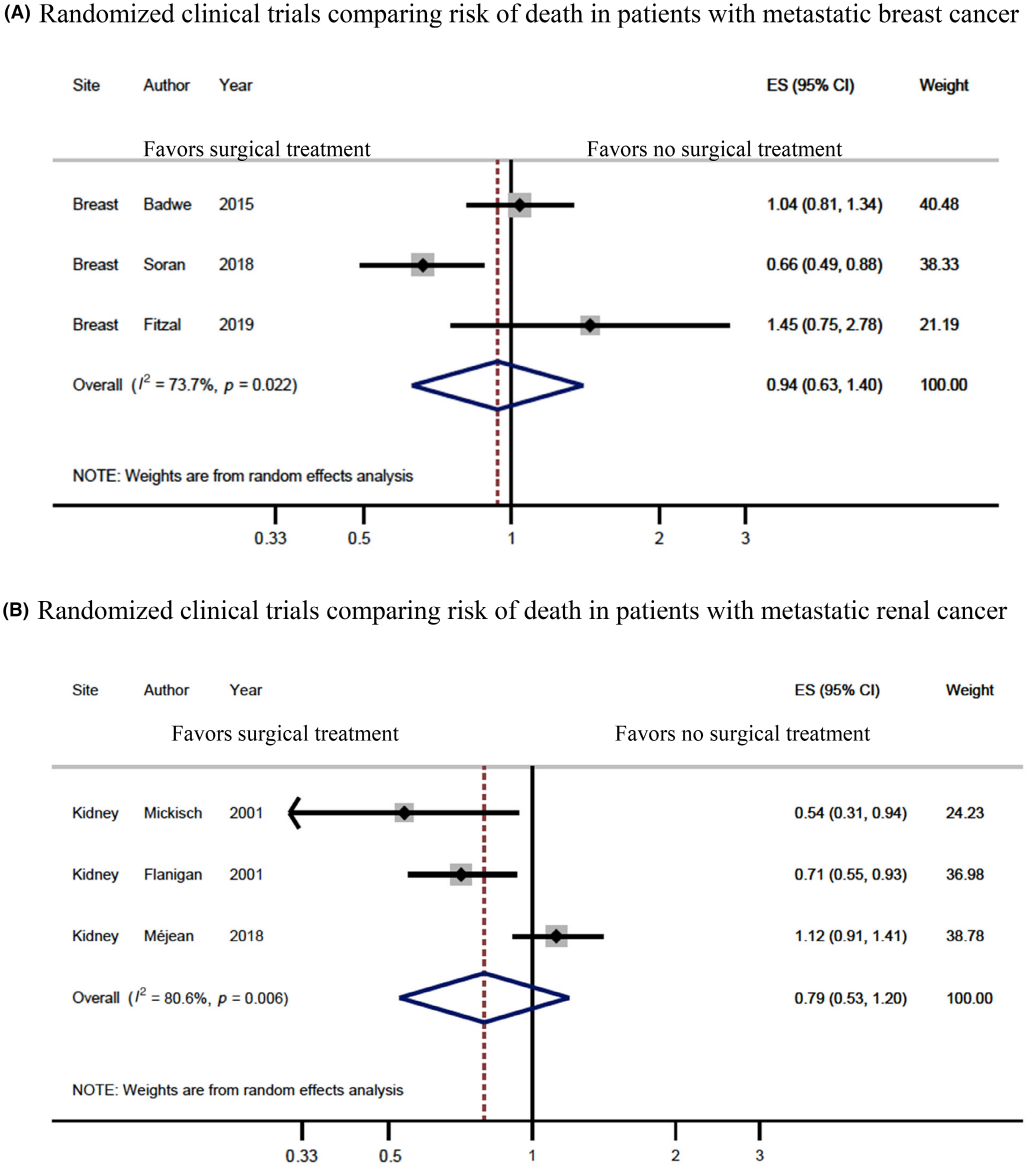
Meta‐analysis results. Hazard ratio (HR) and 95% confidence interval (CI) for death among patients with metastatic breast cancer (A) and metastatic renal cancer (B) who underwent surgical intervention of the primary tumor compared to systemic therapy alone. Results are shown for individual studies and pooled results from meta‐analysis, where the diamond represents the point estimate (95% CI) of the pooled estimate. The solid vertical line is centered at the null, while the dashed line is centered at the pooled HR estimate.

Compared to patients assigned to systemic therapy only, those randomized to locoregional surgery did not have a significant survival advantage in the trials reported by Badwe et al. (HR 1.04, 95% CI 0.81–1.34) or Fitzal et al. (HR 1.45, 95% CI 0.75–2.78) (Figure 2A).^43^, ^44^ However, in the trial reported by Soran et al.,^42^ surgery resulted in a significant survival benefit (HR 0.66, 95% CI 0.49–0.88). Patients in the surgical arm had a median survival of 46 months compared with 37 months for those assigned to systemic therapy only. A pooled hazard demonstrated that surgical intervention and systemic therapy were associated with similar hazard of death as systemic therapy alone (HR 0.94, 95% CI 0.63–1.40). The results of these studies showed considerable statistical heterogeneity, with an *I* ^2^ of 73.7% (*p* = 0.022).

### 3.4 Renal cancer clinical trials

Three studies investigating the role of nephrectomy in metastatic renal cancer included 776 participants, of whom 388 (50.0%) were assigned to surgical intervention followed by systemic therapy and 388 (50.0%) to systemic therapy only.^9^, ^10^, ^45^ All studies required histological confirmation of metastatic renal‐cell carcinoma obtained by biopsy or needle aspiration and excluded patients who had previous systemic therapy or radiation therapy. Overall survival was the primary endpoint for all three studies. Two studies excluded patients who had brain metastases.^10^, ^45^ Patients treated in the studies reported by Flanigan et al.^9^ and Mickisch et al.^10^ received interferon‐alpha‐based systemic therapy, while those enrolled in the study reported by Méjean et al.^45^ in 2018 were treated with sunitinib.

The studies by Flanigan et al.^9^ (HR 0.71, 95% CI 0.55–0.93) and Mickisch et al.^10^ (HR 0.54, 95% CI 0.31–0.94) found that cytoreductive nephrectomy followed by interferon‐based immunotherapy achieved a significant overall survival benefit compared to immunotherapy alone (Figure 2B). On the contrary, Méjean et al.^45^ found that among patients treated with sunitinib, systemic therapy alone was non‐inferior to systemic therapy after cytoreductive nephrectomy (HR 1.12, 95% CI 0.91–1.41). Pooling the results of these studies demonstrated that receiving surgical therapy was not significantly associated with a reduction in the risk of all‐cause mortality compared to systemic therapy alone (HR 0.79, 95% CI 0.53–1.20). However, the studies showed considerable heterogeneity (*I* ^2^ = 80.6%, *p* = 0.006).

### 3.5 Stomach and colorectal cancer clinical trials

Fujitani et al.^46^ randomized patients with advanced gastric cancer and a single non‐curable factor such as a liver, peritoneal, or 16a1/b2 lymph node metastasis to gastrectomy and chemotherapy versus chemotherapy alone. Metastatic lesions were not resected at the time of gastrectomy. Systemic therapy consisted of S‐1 and cisplatin. The study included 175 patients, of whom 89 received surgery and systemic therapy and 86 received systemic therapy alone. The study had a median follow‐up time of 14.5 months. Gastrectomy and systemic therapy resulted in similar overall survival compared with systemic therapy alone (HR 1.09, 95% CI 0.78–1.52).

In 2003, Verwaal et al.^39^ reported a trial that evaluated the impact of cytoreductive surgery and HIPEC on survival among patients with colon or rectal cancer with peritoneal carcinomatosis. Fifty‐four patients underwent aggressive cytoreductive surgery followed by HIPEC followed by systemic therapy, while an additional 51 patients underwent systemic chemotherapy with or without palliative surgery. Patients were followed for an average of 21.6 months, and the median survival was 12.6 months in the systemic therapy arm and 22.3 months in the surgical arm, corresponding to a statistically significant survival benefit of the aggressive cytoreductive surgery with HIPEC compared to systemic therapy alone (HR 0.55, 95% CI 0.32–0.95).

### 3.6 Resection of primary tumor among patients with metastatic cancer in the United States

We identified 152,606 patients who were diagnosed with de novo metastatic cancer in the SEER registry between 2012 and 2016. Among these patients, 129,950 (85%) were diagnosed with one of the following 10 most common metastatic cancers: lung, pancreas, colon, breast, prostate, prostate, ovary, stomach, kidney, rectum, or esophagus (Table 2). The proportion of patients undergoing surgery with distant metastases varied widely cancer type. The highest rates of surgical intervention were in metastatic ovarian (69.4%) and colon cancer (48.3%) while surgery was undertaken rarely for patients with prostate (2.1%), lung (3.3%), and pancreatic cancer (3.7%). Median survival varied considerably between cancer sites, ranging from 6 months for metastatic pancreatic cancer to 39 months for metastatic breast cancer.

**TABLE 2:** Top 10 most common metastatic cancers in the SEER database (2012–2016) and the relevant randomized clinical trials assessing the impact of surgery on survival.

	U.S surveillance epidemiology and end results program, 2012–2016			Randomized controlled trials analysis		
	Patients with distant metastases	Patients with distant metastases undergoing surgery, *n* (%)	Median survival, months (95% CI)	Relevant randomized trials?	Pooled hazard ratio (95% CI)^a^	Bias assessment
Lung	58,924	1938 (3.3)	10 (10–10)	No	–	–
Pancreas	14,488	545 (3.8)	6 (6–6)	No	–	–
Colon	11,459	5538 (48.3)	19 (18–20)	Yes	0.55 (0.32–0.95)	Low
Breast	8815	1957 (22.2)	39 (38–41)	Yes	0.94 (0.63–1.40)	Low or medium
Prostate	8698	181 (2.1)	33 (32–34)	No	–	–
Ovary	8112	5627 (69.4)	38 (36–40)	No	–	–
Stomach	5978	663 (11.1)	9 (8–9)	Yes	1.09 (0.78–1.52)	Low
Kidney	4979	1704 (34.2)	12 (11–13)	Yes	0.79 (0.53–1.20)	Low
Rectum	4965	1545 (31.1)	24 (23–25)	Yes	0.55 (0.32–0.95)	Low
Esophagus	3532	0 (0.0)	9 (9–10)	No	–	–

^a^ Hazard ratio <1 favors surgical treatment.

The frequency of primary site surgery by cancer site did not appear to correspond with the existence of randomized trial nor the average prognosis associated with the cancer. For example, patients with metastatic ovarian cancer had the highest rates of surgical intervention compared to any other cancer site, despite no randomized trials comparing surgery and systemic therapy with chemotherapy alone. However, only 31.1%–48.3% of patients with metastatic colon or rectal cancer received surgery, even though the study by Verwaal et al. showed a statistically significant survival benefit of aggressive cytoreductive surgery with HIPEC compared to systemic therapy alone. For breast cancer and renal cell carcinoma, which had the highest number of relevant randomized clinical trials and conflicting findings, 22.2%–34.2% of patients received surgery. The sites of cancer with the lowest rates of surgical intervention, like prostate, lung, and pancreas, lacked randomized clinical trials comparing surgical intervention to systemic therapy in the metastatic setting.

## 4 DISCUSSION

In this study evaluating current evidence and practice with respect to cancer‐directed surgery among patients with 10 common metastatic cancers, we identified eight randomized trials that evaluated the efficacy of surgery in breast, renal, stomach, and colorectal cancer. In breast and renal cancer, results of the randomized trials were heterogenous, with some but not all studies suggesting benefit for locoregional surgery. Pooled analyses found that there was no consistent evidence of benefit for either site. One study evaluating gastrectomy in metastatic stomach cancer found no evidence of benefit, and a small trial suggested that surgery and HIPEC might be beneficial in colorectal cancer with peritoneal metastasis. Among patients diagnosed with a metastatic cancer in the United States, the existence of a positive randomized trial did not appear to be the most important driver of the utilization of primary site surgery. While surgical intervention was performed somewhat frequently for metastatic breast, renal, and colon cancer, the highest rates of surgical intervention were seen in ovarian cancer patients despite the absence of randomized trials. Surgery was rare in metastatic prostate, lung, and pancreatic cancer, all of which lacked randomized trials evaluating its impact.

Our results are consistent with prior systematic review and meta‐analyses across different cancer sites. A Cochrane meta‐analysis of two randomized trials evaluating surgery in metastatic breast cancer demonstrated unclear benefit of surgery on overall survival, driven by low quality evidence.^16^ In our analysis, we included three breast cancer trials, including one additional study and an original study that provided the complete data from the abstract included in the Cochrane review. All of the three studies were considered low to medium risk of bias using the updated RoB2 bias assessment scoring system. Within the renal cancer literature, two prior meta‐analyses that included retrospective observational studies concluded that nephrectomy was effective at improving overall survival in patients with metastatic renal carcinoma.^17^, ^47^ Our analysis of the three available randomized trials found no clear evidence of benefit and significant unexplained heterogeneity among trial results. Similarly, a meta‐analysis of observational studies found a slight benefit for surgery among patients with metastatic colon cancer, which was mirrored by the one randomized trial we found in our study.^18^

Our study highlights substantial knowledge gaps in the literature addressing the role of surgery in metastatic cancer. Among the 10 most common de novo metastatic cancers, no randomized trials compared surgery and systemic therapy versus systemic therapy alone in lung, pancreatic, prostate, ovarian, and esophageal cancers. However, surgical treatment for de novo metastatic ovarian cancer is standard despite a lack of randomized trials substantiating this practice. This finding highlights the role that non‐randomized evidence plays in shaping real‐world practice. For certain cancer sites, clinicians' beliefs about the role of upfront surgery may be so fixed that a randomized trial may seem unnecessary or unethical, either because of perceived benefits or harms. In general, it appears that both randomized trials of surgery and real‐world utilization of surgical procedures are less common in metastatic cancers with median survivals of less than 1 year. In addition to prognosis, the morbidity of an operation required to excise the primary tumor may also influence site‐specific utilization of surgery in the metastatic setting. Further research is needed to understand the underlying reasons for variation in the utilization of surgery across cancer sites and to estimate its effectiveness.

Randomized clinical trials are the least biased means of evaluating the effect of surgery on survival in metastatic cancer. Because patients who undergo surgical intervention in a real‐world setting are selected based on health status and perceived benefit of surgery, comparative effectiveness studies based on observational data are at risk for bias due to unmeasured confounding, immortal time bias, and confounding by indication. Nonetheless, the small number of randomized trials identified by our systematic review makes apparent the significant difficulty of completing experimental studies. In theory, quasi‐experimental observational methods that can estimate unbiased treatment effects of surgery among patients with metastatic cancers in the presence of unmeasured confounders might provide insights in this field, though the validity of such measure in this context remains unknown.^48^

### 4.1 Limitations

Our study contextualizes real‐world surgical practice in the setting of metastatic cancer within the existing randomized evidence base. However, our study should be considered acknowledging several limitations. The considerable heterogeneity of the studies presented limit the ability to identify broad recommendations about surgical interventions for de novo metastatic disease. We were also limited by the number of randomized clinical trials that were available for the analysis. Due to the limited number of trials, we were unable to tease out the impact of surgery for the primary site only compared with surgery to primary and metastatic disease sites. For the same reason, we were unable to evaluate whether surgery is more effective in patients with oligometastatic disease compared to those with more widely metastatic cancer. The extent of metastasis and the ability of the surgeon to completely resect both local and metastatic tumors may play an important role in contributing to the efficacy of surgery in this setting. Finally, despite a rigorous search of the primary and gray literature designed by a librarian who specializes in systematic reviews, it is possible that some randomized trials were not included in our analysis.

## 5 CONCLUSIONS

This systematic review and meta‐analysis of high‐quality randomized clinical trials suggests that some individuals with de novo metastatic disease may benefit from cancer‐directed surgical intervention, but that the available trial data are too heterogeneous to draw general conclusions and too sparse to identify which patients are likely to benefit from this treatment approach. The use of surgery among patients with metastatic cancer varies significantly between cancer sites, and this variation does not appear to be explained the findings of randomized trials.

## AUTHOR CONTRIBUTIONS

**Mary Kathryn Abel:** Conceptualization (supporting); data curation (lead); formal analysis (equal); investigation (equal); methodology (equal); resources (equal); writing—original draft (lead); writing—review and editing (lead). **Ellen L. Myers:** Conceptualization (supporting); data curation (equal); formal analysis (equal); methodology (equal); resources (equal); writing—original draft (supporting); writing—review and editing (equal). **Ellen Minkin:** Conceptualization (supporting); data curation (equal); formal analysis (equal); methodology (supporting); writing—original draft (supporting); writing—review and editing (equal). **Peggy Tahir:** Conceptualization (supporting); data curation (equal); formal analysis (equal); methodology (equal); writing—review and editing (equal). **Alex B. Haynes:** Conceptualization (supporting); resources (equal); writing—review and editing (equal). **Jason D. Wright:** Conceptualization (supporting); resources (equal); writing—review and editing (equal). **J. Alejandro Rauh‐Hain:** Conceptualization (supporting); resources (equal); writing—review and editing (equal). **Alexander Melamed:** Conceptualization (lead); data curation (equal); formal analysis (equal); funding acquisition (equal); investigation (equal); methodology (equal); resources (equal); software (equal); writing—original draft (equal); writing—review and editing (equal).

## Supporting information


Data S1:

## Data Availability

Data are available from the authors upon reasonable request.
