# Supplementary material for: Cancer‐directed surgery in patients with metastatic cancer: A systematic review and meta‐analysis of randomized evidence
Source: Cancer Med. 2023 Jun 13;12(13):14072–83. doi: 10.1002/cam4.6061 (PMC10358262; doi:10.1002/cam4.6061)
Supplement: Supplementary file 1 — Data S1: [file CAM4-12-14072-s001.docx]

eTable1: Search Strings

| Database | Search Strings |
| --- | --- |
| PubMed (January 1, 1995 and June 17, 2020) | ("Lung Neoplasms/surgery"[Mesh] OR "Pancreatic Neoplasms/surgery"[Mesh] OR "Ovarian Neoplasms/surgery"[Mesh] OR "Breast Neoplasms/surgery"[Mesh] OR "Prostatic Neoplasms/surgery"[Mesh] OR "Stomach Neoplasms/surgery"[Mesh] OR "Kidney Neoplasms/surgery"[Mesh] OR "Colonic Neoplasms/surgery"[Mesh] OR "Esophageal Neoplasms/surgery"[Mesh] OR "Rectal Neoplasms/surgery"[Mesh]) OR ((“lung cancer”[tiab] OR “lung neoplasms”[tiab] OR “pancreatic cancer”[tiab] OR “pancreatic neoplasms”[tiab] OR “ovarian cancer”[tiab] OR “ovarian neoplasms”[tiab] OR “breast cancer”[tiab] OR “breast neoplasms”[tiab] OR “prostate cancer”[tiab] OR “prostatic cancer”[tiab] OR “prostatic neoplasms”[tiab] OR “stomach cancer”[tiab] OR “stomach neoplasms”[tiab] OR “kidney cancer”[tiab] OR “kidney neoplasms”[tiab] OR “colon cancer”[tiab] OR “colonic cancer”[tiab] OR “colonic neoplasms”[tiab] OR “esophageal cancer”[tiab] OR “esophageal neoplasms”[tiab] OR “rectal cancer”[tiab] OR “rectal neoplasms”[tiab]) AND surgery)  AND  (“wedge resection” OR “segmental resection” OR segmentectomy OR lobectomy OR bilobectomy OR pneumonectomy OR "Pneumonectomy"[Mesh] OR “distal pancreatectomy” OR “total pancreatectomy” OR "Pancreatectomy"[Mesh] OR pancreatoduodenectomy OR whipple OR “cytoreductive surgery” OR cytoreduction OR "Cytoreduction Surgical Procedures"[Mesh] OR debulking OR lumpectomy OR “breast conservation surgery” OR “segmental mastectomy” OR mastectomy OR “Mastectomy”[Mesh] OR “radical mastectomy” OR “skin sparing mastectomy” OR “total skin sparing mastectomy” OR “oncoplastic reduction mammoplasty” OR prostatectomy OR "Prostatectomy"[Mesh] OR “subtotal gastrectomy” OR “total gastrectomy” OR "Gastrectomy"[Mesh] OR “endoscopic resection” OR nephrectomy OR "Nephrectomy"[Mesh] OR "Kidney Pelvis/surgery"[Mesh] OR “partial colectomy” OR “subtotal colectomy” OR hemicolectomy OR “total colectomy” OR "Colectomy"[Mesh] OR “total proctocolectomy” OR colectomy OR esophagectomy OR "Esophagectomy"[Mesh] OR proctosigmoidectomy OR proctectomy OR "Proctectomy"[Mesh] OR “total mesorectal excision” OR “low anterior resection” OR “abdominoperineal resection” OR “pelvic exenteration” OR "Pelvic Exenteration"[Mesh] OR colostomy OR "Colostomy"[Mesh])  AND  (("Neoplasm Metastasis/surgery"[Mesh] OR “secondary”[subheading] OR metastatic OR metastasis OR metastases OR “stage IV” OR advanced) NOT “locally advanced”)  AND  (“survival analysis” OR "Survival Analysis"[Mesh] OR mortality OR “Mortality”[mesh] OR “mortality”[subheading])  AND  ("randomized controlled trial"[Publication Type] OR "randomized controlled trials as topic"[MeSH Terms] OR "randomized controlled trial"[All Fields] OR "randomised controlled trial"[All Fields]) |
| Web of Science (January 1, 1995 and June 17, 2020) | ((lung cancer OR lung neoplasms OR pancreatic cancer OR pancreatic neoplasms OR ovarian cancer OR ovarian neoplasms OR breast cancer OR breast neoplasms OR prostate cancer OR prostatic cancer OR prostatic neoplasms OR stomach cancer OR stomach neoplasms OR kidney cancer OR kidney neoplasms OR colon cancer OR colonic cancer OR colonic neoplasms OR esophageal cancer OR esophageal neoplasms OR rectal cancer OR rectal neoplasms) AND surgery)  AND  (“wedge resection” OR “segmental resection” OR segmentectomy OR lobectomy OR bilobectomy OR pneumonectomy OR “distal pancreatectomy” OR “total pancreatectomy” OR "Pancreatectomy" OR pancreatoduodenectomy OR whipple OR “cytoreductive surgery” OR cytoreduction OR debulking OR lumpectomy OR “breast conservation surgery” OR “segmental mastectomy” OR mastectomy OR “radical mastectomy” OR “skin sparing mastectomy” OR “total skin sparing mastectomy” OR “oncoplastic reduction mammoplasty” OR prostatectomy OR “subtotal gastrectomy” OR “total gastrectomy” OR "Gastrectomy" OR “endoscopic resection” OR nephrectomy OR “partial colectomy” OR “subtotal colectomy” OR hemicolectomy OR “total colectomy” OR “total proctocolectomy” OR colectomy OR esophagectomy OR proctosigmoidectomy OR proctectomy OR “total mesorectal excision” OR “low anterior resection” OR “abdominoperineal resection” OR “pelvic exenteration” OR colostomy)  AND  ((metastatic OR metastasis OR metastases OR “stage IV” OR advanced) NOT “locally advanced”)  AND  (“survival analysis” OR mortality)  AND  (randomized controlled trial OR RCT) |
| Embase (January 1, 1995 and June 17, 2020) | ('lung cancer'/exp/dm_su OR 'pancreas cancer'/exp/dm_su OR 'breast cancer'/exp/dm_su OR 'prostate cancer'/exp/dm_su OR 'stomach cancer'/exp/dm_su OR 'kidney cancer'/exp/dm_su OR 'colon cancer'/exp/dm_su OR 'esophagus cancer'/exp/dm_su OR 'rectum cancer'/exp/dm_su) OR (('lung cancer':ab,ti OR 'lung tumor':ab,ti OR 'pancreas cancer':ab,ti OR 'pancreas tumor':ab,ti OR ‘pancreatic cancer’:ab,ti OR 'ovary cancer':ab,ti OR 'ovary tumor':ab,ti OR ‘ovarian cancer’:ab,ti OR 'breast cancer':ab,ti OR 'breast tumor':ab,ti OR 'prostate cancer':ab,ti OR 'prostate tumor':ab,ti OR 'stomach cancer':ab,ti OR 'stomach tumor':ab,ti OR 'kidney cancer':ab,ti OR 'kidney tumor':ab,ti OR 'colon cancer':ab,ti OR 'colon tumor':ab,ti OR 'esophagus cancer':ab,ti OR 'esophagus neoplasms':ab,ti OR 'esophagus tumor':ab,ti OR ‘esophageal cancer’:ab,ti OR 'rectum cancer':ab,ti OR 'rectum tumor':ab,ti OR ‘rectal cancer’:ab,ti) AND surgery)  AND  ('wedge resection'/exp OR 'wedge resection' OR 'segmental resection' OR 'segmentectomy'/exp OR segmentectomy OR 'lobectomy'/exp OR lobectomy OR 'bilobectomy'/exp OR bilobectomy OR pneumonectomy OR 'lung resection'/exp OR 'lung resection' OR 'distal pancreatectomy'/exp OR 'distal pancreatectomy' OR 'total pancreatectomy' OR 'pancreatectomy'/exp OR pancreatectomy OR 'pancreaticoduodenectomy'/exp OR pancreaticoduodenectomy OR whipple OR 'cytoreductive surgery'/exp OR 'cytoreductive surgery' OR 'cytoreduction'/exp OR cytoreduction OR debulking OR 'lumpectomy'/exp OR lumpectomy OR 'breast conservation surgery'/exp OR 'breast conservation surgery' OR 'partial mastectomy'/exp OR 'partial mastectomy' OR ‘segmental mastectomy’ OR 'mastectomy'/exp OR mastectomy OR 'radical mastectomy'/exp OR 'radical mastectomy' OR 'skin sparing mastectomy'/exp OR 'skin sparing mastectomy' OR 'total skin sparing mastectomy'/exp OR 'total skin sparing mastectomy' OR 'oncoplastic reduction mammoplasty' OR 'prostatectomy'/exp OR prostatectomy OR 'subtotal gastrectomy'/exp OR 'subtotal gastrectomy' OR 'total stomach resection'/exp OR 'total stomach resection' OR 'total gastrectomy' OR 'gastrectomy'/exp OR gastrectomy OR 'endoscopic resection' OR 'nephrectomy'/exp OR nephrectomy OR 'partial colectomy'/exp OR 'partial colectomy' OR 'colon resection'/exp OR 'colon resection' OR 'subtotal colectomy' OR 'hemicolectomy'/exp OR hemicolectomy OR 'total colectomy' OR 'total colon resection'/exp OR 'total colon resection' OR 'colectomy'/exp OR colectomy OR 'total proctocolectomy'/exp OR 'total proctocolectomy' OR 'esophagectomy'/exp OR esophagectomy OR 'esophagus resection'/exp OR 'esophagus resection' OR proctosigmoidectomy OR 'proctectomy'/exp OR proctectomy OR 'rectum resection'/exp OR 'rectum resection' OR 'total mesorectal excision'/exp OR 'total mesorectal excision' OR 'low anterior resection'/exp OR 'low anterior resection' OR 'abdominoperineal resection' OR 'rectum abdominoperineal resection'/exp OR 'rectum abdominoperineal resection' OR 'pelvis exenteration'/exp OR 'pelvis exenteration' OR 'colostomy'/exp OR colostomy)  AND  (('metastasis'/exp/dm_su OR metastatic OR 'metastasis'/exp OR metastasis OR metastases OR 'stage IV' OR advanced) NOT 'locally advanced')  AND  ('survival analysis'/exp OR 'survival analysis' OR 'cancer mortality'/exp OR 'cancer mortality')  AND  ('randomized controlled trial (topic)'/exp OR 'randomized controlled trial (topic)' OR 'randomized controlled trial'/exp OR 'randomized controlled trial' OR RCT) |

eTable 2: Primary cancer sites, description of surgery, and associated surgical codes from the Surveillance, Epidemiology, and End Results (SEER) program

| **Primary Site (ICD-0-3 codes)** | **Description of Surgery and Associated Codes** |
| --- | --- |
| Breast (C50.0-C50.9) | Partial mastectomy (20-24), total (simple mastectomy (40-42), modified radical mastectomy (50-59, 63) |
| Colon (C18.0-C18.9) | Partial colectomy segmental resection (30-32), subtotal colectomy/hemicolectomy (40-41), total colectomy (50-51), total proctocolectomy (60-61), colectomy or coloproctocolectomy with resection of contiguous organs (70), colectomy NOS (80) |
| Esophagus (C15.0-C15.9) | Partial esophagectomy (30), total esophagectomy, NOS (40), esophagectomy NOS with laryngectomy and/or gastrectomy, NOS (50-55), esophagectomy, NOS (80) |
| Kidney (C64.9, C65.9) | Partial or subtotal nephrectomy or partial ureterectomy (30), complete/total/simple nephrectomy (40), radical nephrectomy (50), any nephrectomy in continuity with the resection of other organ(s) (70), nephrectomy or ureterectomy, NOS (80) |
| Lung (C34.0-C34.9) | Excision or resection of less than one lobe, NOS (20-25), resection of at least one lobe or bilobectomy, but less than the whole lung (partial pneumonectomy, NOS) (30, 33), lobe or bilobectomy extended, NOS (45-48), pneumonectomy, NOS (55, 56), extended pneumonectomy (65-66), extended radical pneumonectomy (70), resection of lung, NOS (80) |
| Ovary (C56.9) | Total removal of tumor or (single) ovary, NOS (25-28), unilateral (salpingo-)oophorectomy; unknown if hysterectomy done (35-37), bilateral (salpingo-)oophorectomy; unknown if hysterectomy done (50-52), unilateral or bilateral (salpingo-)oophorectomy with omentectomy, NOS (55-57), debulking; cytoreductive surgery, NOS (60-63), pelvic exenteration, NOS; anterior, posterior, total, or extended (70-74), (salpingo-)oophorectomy, NOS (80) |
| Pancreas (C25.0-C25.9) | Partial pancreatectomy, NOS (30), local or partial pancreatectomy and duodenectomy (35-37), total pancreatectomy (40), total pancreatectomy and subtotal gastrectomy or duodenectomy (60), extended pancreatodudenectomy (70), pancreatectomy, NOS (80) |
| Prostate (C61.9) | Subtotal, segmental, or simple prostatectomy, which may leave all or part of the capsule intact (30), radical prostatectomy, NOS; total prostatectomy, NOS (50), prostatectomy with resection in continuity with other organs; pelvic exenteration (70); prostatectomy, NOS (80) |
| Rectum (C20.9),  Rectosigmoid Junction (C19.9) | Wedge or segmental resection; partial proctectomy, NOS (30), pull through with sphincter preservation (40), total proctectomy (50), total proctocolectomy, NOS (60), proctectomy or proctocolectomy with resection in continuity with other organs; pelvic exenteration (70), proctectomy, NOS (80)  Wedge or segmental resection; partial proctosigmoidectomy, NOS (30-31), pull through with sphincter preservation (colo-anal anastomosis) (40), total proctectomy (50), total colectomy (51), total colectomy with ileostomy, NOS (55-57), total proctocolectomy, NOS (60, 65-66), colectomy or proctocolectomy resection in continuity with other organs; pelvic exenteration (70), colectomy NOS; proctectomy, NOS (80) |
| Stomach (C16.0-C16.9) | Gastrectomy, NOS (partial, subtotal, hemi-) (30-33), near-total or total gastrectomy (40-42), gastrectomy, NOS with removal of a portion of the esophagus (50-52), gastrectomy with resection in continuity with the resection of other organs, NOS (60-63), gastrectomy, NOS (80) |

eTable 3: Risk of bias assessments using the Revised Cochrane Risk-of-Bias Tool for Randomized Trials (RoB2)^1^


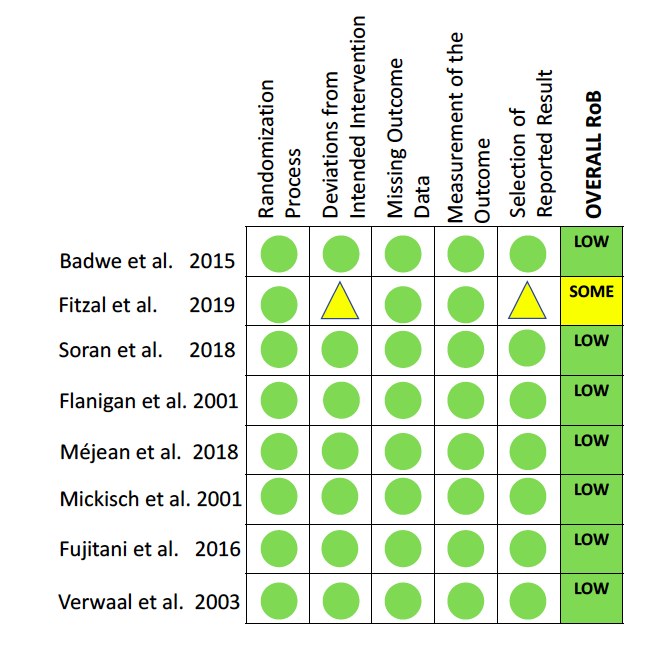


^1^Thresholds for converting RoB2 scores into overall risk of bias assessment:

**Low**: low risk of bias for all domains

**Some**: some risk in at least one domain but not high risk in any domain

**High**: high risk in at least one domain or some concerns for multiple domains in a way that substantially lowers confidence in the result
